# Detection of Hg^2+^ Using a Dual-Mode Biosensing Probe Constructed Using Ratiometric Fluorescent Copper Nanoclusters@Zirconia Metal-Organic Framework/*N*-Methyl Mesoporphyrin IX and Colorimetry G-Quadruplex/Hemin Peroxidase-Mimicking G-Quadruplex DNAzyme

**DOI:** 10.34133/bmef.0078

**Published:** 2024-12-17

**Authors:** Shikha Jain, Monika Nehra, Neeraj Dilbaghi, Ganga Ram Chaudhary, Sandeep Kumar

**Affiliations:** ^1^ Department of Bio-nanotechnology, College of Biotechnology, CCS Haryana Agricultural University (CCSHAU), Hisar-Haryana 125004, India.; ^2^ Department of Chemistry & Center of Advanced Studies in Chemistry, Panjab University, Chandigarh 160014, India.; ^3^ Department of Mechanical Engineering, University Institute of Engineering and Technology, Panjab University, Chandigarh 160014, India.; ^4^ Department of Bio and Nano Technology, Guru Jambheshwar University of Science and Technology, Hisar-Haryana 125001, India.; ^5^ Department of Physics, Punjab Engineering College (Deemed to be University), Chandigarh 160012, India.

## Abstract

Mercury (Hg^2+^) has been recognized as a global pollutant with a toxic, mobile, and persistent nature. It adversely affects the ecosystem and human health. Already developed biosensors for Hg^2+^ detection majorly suffer from poor sensitivity and specificity. Herein, a colorimetric/fluorimetric dual-mode sensing approach is designed for the quantitative detection of Hg^2+^. This novel sensing approach utilizes nanofluorophores, i.e., fluorescent copper nanoclusters-doped zirconia metal-organic framework (CuNCs@Zr-MOF) nanoconjugate (blue color) and *N*-methyl mesoporphyrin IX (NMM) (red color) in combination with peroxidase-mimicking G-quadruplex DNAzyme (PMDNAzyme). In the presence of Hg^2+^, dabcyl conjugated complementary DNA with T–T mismatches form the stable duplex with the CuNCs@Zr-MOF@G-quadruplex structure through T–Hg^2+^–T base pairing. It causes the quenching of fluorescence of CuNCs@Zr-MOF (463 nm) due to the Förster resonance energy transfer (FRET) system. Moreover, the G-quadruplex (G4) structure of the aptamer enhances the fluorescence emission of NMM (610 nm). Besides this, the peroxidase-like activity of G4/hemin DNAzyme offers the colorimetric detection of Hg^2+^. The formation of duplex with PMDNAzyme increases the catalytic activity. This novel biosensing probe quantitatively detected Hg^2+^ using both fluorimetry and colorimetry approaches with a low detection limit of 0.59 and 36.3 nM, respectively. It was also observed that the presence of interfering metal ions in case of real aqueous samples does not affect the performance of this novel biosensing probe. These findings confirm the considerable potential of the proposed biosensing probe to screen the concentration of Hg^2+^ in aquatic products.

## Introduction

Mercury ions (Hg^2+^), increasingly recognized as highly toxic heavy metal ions, pose a severe hazard to health and environment [1]. All forms of mercury are poisonous, whether inorganic, organic, or elemental [2]. In addition to the coal-fired power plants, the major contributor in environmental Hg waste is industries dealing with electronic and electrical devices such as thermostats, fluorescent lights, certain batteries, automotive parts, and liquid crystal display screens. Its long-term persistence in environment and further bioaccumulation severely threatens human health and other organisms. Among hazardous metals, it is ranked third by United States Environmental Protection Agency (EPA). It mainly affects the brain’s central nervous system and causes acute renal failure, autoimmune, Alzheimer’s, and Parkinson’s diseases. Some chemical forms of Hg (like HgCl_2_) have also proven to be carcinogenic [3,4]. Moreover, its high solubility causes its quick spread through cell membranes and blood to the tissues, leading to speech and hearing loss, permanent damage to the brain, and cognitive impairment.

The world is on the brink of disaster due to unabated mercury imports and a pollution problem that has already reached catastrophic proportions. Therefore, monitoring of Hg exposure is a must to protect the human health and the environment. At present, most popular techniques deployed to detect Hg^2+^ include inductively coupled plasma–mass spectrometry (ICP-MS) [5], atomic absorption spectrometry [6], and electrochemical analysis [7]. However, application of these technologies for on-site detection is limited because of their complex sample preparation, professional executors, and expensive equipment. This motivates the researchers to fabricate fast, cost-effective, and real-time biosensors for the sensitive determination of Hg^2+^.

Various novel biosensors based on surface-enhanced Raman scattering (SERS) [8], electrochemiluminescence [9], electrochemistry [10], colorimetry [11], and fluorimetry [12–14] approaches have been developed for the detection of heavy metal ions. Among all, fluorescent and colorimetric techniques are employed widely in designing real-time biosensors due to their fast response, naked-eye readout, simple instrumentation, promising analytical performance, and high sensitivity with distinct color variation [15,16]. Moreover, ratiometric fluorometry (that utilizes two or more emission bands) improves the accuracy of measurement and also eliminates signal fluctuations [17,18]. Therefore, it will be more valuable in practice to develop ratiometric fluorescence methods to detect Hg^2+^. Fluorescent materials, like organic dyes, metallic nanoparticles, and quantum dots (QDs), are becoming promising candidates for biosensing and imaging applications. In the last decade, nanofluorophore-tagged oligoprobes have been reported as simple and sensitive detection methods. Li et al. [19] utilized upconversion nanoclusters and thymine (T)-enriched aptamer to develop a sensing strategy based on “turn-on” fluorescent detection of Hg^2+^. In this work, polydopamine nanoparticles (PDANPs) were used as quencher. In the presence of metal ion (Hg), the aptamer forms a hairpin-like structure, causing release of PDANPs, which results in recovery of fluorescence. Zhou et al. [20] utilized gold nanoparticle (AuNP)-decorated Ti_3_C_2_T*_X_* nanohybrid as a novel nanozyme to develop a colorimetric method for sensitive sensing of Hg^2+^. There have been great advances in the detection of Hg^2+^ using fluorescent and colorimetric methods, but most of these studies focus on a single sensing modality. This choice cannot eliminate the interference from other factors like biological entities, instrumental efficiency, and operating conditions.

Surprisingly, the idea of integrating different modalities is a promising strategy to immensely enhance the sensing performance [21]. Dual-mode sensing commendably inherits the strength of individual method and significantly enhances the accuracy and reliability of analysis [22]. Dual-mode sensors get benefited from self-calibration and anti-interference ability. Therefore, a fluorometric and colorimetric dual-mode sensor platform is significantly attractive, where fluorescent analysis enhances the detection’s sensitivity and colorimetry provides naked-eye readouts [23]. For instance, Kaewnok et al. [24] presented a dual-mode sensor utilizing rhodamine-incorporated blue silver nanoparticles (RS+b-AgNPs) for the fluorometric/colorimetric detection of Hg^2+^. Fan et al. [25] reported a novel dual-channel (colorimetric/fluorometric) sensing system for the detection of ampicillin on the basis of the fluorescent silver nanocluster (AgNC)-tagged aptamer and MoSe_2_ nanosheets. The AgNC–Apt conjugate absorbed on nanosheets causes Förster resonance energy transfer (FRET) between AgNCs and MoSe_2_. In addition to desorption of the aptamer from nanosheets in the presence of ampicillin, it also triggers the peroxidase activity of MoSe_2_, leading to colorimetric response. Unfortunately, there is a limited research work on dual-mode biosensor for selective and sensitive sensing of Hg^2+^. The development of dual-mode strategies with robust analytical performance is therefore highly desired for Hg^2+^ monitoring.

DNAzymes, being artificial DNA sequences, have got tremendous research attention as biocatalyst due to their excellent properties such as high catalytic activities, easy synthesis, specificity, and thermal stability [26,27]. In particular, G-quadruplexes (G4) are a kind of G-rich DNAzymes, forming G tetrads in the presence of hemin and also exhibiting a peroxidase-mimicking activity. A wide variety of biosensing platforms utilizing G4 as sensing probes for various biomolecules analysis has been developed [28,29]. In addition to the peroxidase-mimicking activity of nucleic acid, its conjugation with fluorescent nanomaterials (e.g., QDs, metal nanoclusters, and upconversion nanoparticles) and quencher can offer fluorescence resonance energy transfer (FRET) effect, serving as a ratiometric fluorescence probe. Therefore, a few biosensors utilizing the FRET process have been developed using G4 for sensing of different analytes [30,31].

Metal nanoclusters, a new kind of fluorescent nanomaterials, are promising alternatives to traditional organic dyes and QDs due to their novel properties, such as easy synthesis, small size, photostability, low toxicity, and good biocompatibility [32,33]. Among variety of nanoclusters, copper nanoclusters (CuNCs) are the most frequently used nanomaterial due to their low cost, abundance, good water solubility, nontoxic characteristics, small particle size, and high quantum yield. Specifically, the ratiometric fluorescent sensors have been constructed by combining them with other fluorescent materials. Many porphyrin dyes including thioflavin T and N-methylmesoporphyrin IX specifically bind to G4, generating multi-fold enhanced fluorescence. Therefore, nanoclusters/porphyrin dyes can be ideal substrate to design ratiometric fluorescent biosensors. Utilizing this principle, Ma et al. [34] employed G4/*N*-methyl mesoporphyrin IX (NMM) and p-nitrophenol (PNP) as a fluorophore and quencher, respectively, to generate fluorescent changes. Meanwhile, the yellow color of PNP itself may be used as a color signal. Here, yellow PNP is produced by dephosphorylation of para-nitrophenyl phosphate (PNPP) using alkaline phosphatase (ALP). G4/NMM fluorescence and PNP color change result in a fluorescence/colorimetric dual-mode immunoassay of zearalenone (ZEN). Guo and coworkers [35] reported ratiometric fluorescent detection of ochratoxin A (OTA) utilizing CuNCs and NMM/G4-DNA. OTA (target analyte) had opposite effects on the fluorescence of both CuNCs and NMM. Nowadays, these dual-mode sensing systems, combining a ratiometric FRET process and G4/hemin’s peroxidase-mimicking property, have become more fascinating for selective and sensitive metal ion detection.

Herein, we present a G4-based fluorescent and colorimetric dual-mode sensing platform for Hg^2+^ detection through combination of the CuNCs-doped zirconia metal-organic framework (CuNCs@Zr-MOF)/NMM and the peroxidase activity of G4 aptamer sequences. The G4 sequence serves as both a recognition element and a signal producer, while CuNCs@Zr-MOF/NMM acts as a fluorescent color generator. The sensing probe is composed of two rationally designed complementary oligo probes: one is a cDNA-dabcyl quencher strand, and the other is a G4 peroxidase-mimicking G-quadruplex DNAzyme (PMDNAzyme) recognition strand. In the presence of Hg^2+^, PMDNAzyme hybridizes to cDNA through the formation of “T–Hg^2+^–T” pairing, forming stable duplex. This stable hybridization induces FRET effect between dabcyl and CuNCs@Zr-MOF, causing a significant quenching of fluorescence emission at 463 nm. Meanwhile, the G4 PMDNAzyme–NMM complex exhibits fluorescence enhancement at 610 nm. These opposite trends of change in fluorescence with Hg^2+^ ions facilitate signal amplification, improved sensitivity, and accuracy in comparison to single-emission fluorescent sensors. Moreover, the formation of DNA duplex also enhances the peroxidase catalytic activity of G4 PMDNAzyme in the presence of hemin. As a result of the peroxidase-like activity, it catalyzes the oxidation of 3,3ʹ,5,5ʹ-tetramethylbenzidine (TMB) in the presence of H_2_O_2_. Therefore, Hg^2+^ can easily be recognized by this fluorometric/colorimetric dual-mode sensing method. Noteworthy, majority of the currently published literature for Hg^2+^centered on a single detection mode, which is vulnerable to inaccurate results due to the background fluorescence, resulting from interfering contaminants in the environment.

## Materials and Methods

### Reagents

Zirconyl chloride [Zr (Cl)_2_·6H_2_O], 2-aminoterepathalic acid, 4-(2-hydroxyethyl) piperazine-1-ethanesulfonic acid (HEPES) sodium salt, copper nitrate (CuNO_3_.2H_2_O), sodium borohydride (NaBH_4_), NMM, glutaraldehyde, and 2-(N-morpholino) ethanesulfonic acid (MES) sodium salt were obtained from Sigma-Aldrich. Hemin, potassium chloride (KCl), sodium chloride, *N*,*N*-dimethyl formamide, acetic acid (CH_3_COOH), hydrogen peroxide (H_2_O_2_), and standard solution of Hg (1000 μg/ml in 1 M HNO_3_) were acquired form Central Drug House (P) Ltd., New Delhi, India. The quencher-labeled oligomer sequences selected from the literature are as follows:

EAD2 aptamer: 5′-TCGTTTCTCTCTCCAACTGGGAGGGAGGGAGGGA-3′

c-DNA oligomer: 5′-TTGGAGAGAGTTTCGA-3′-dabcyl

The aptamers used in this work were synthesized and (high-performance liquid chromatography) purified by Aritech Chemazone Pvt. Ltd., Kurukshetra, Haryana (India). The dabcyl quencher was preferably used for the oligonucleotide sequences’ labeling at the 3′-end of the complementary strand. All acquired chemicals were of analytical grade and utilized without any kind of purification. Nanosep spin filters were procured from Merck. Double-distilled water was used for all experiments.

### Instrumentation

The synthesized samples were fully characterized by several microscopy and spectroscopy techniques. Zetasizer Nano ZS90 (Malvern) was utilized to examine the zeta potentials, hydrodynamic diameters, and polydispersity indices of the samples. Ultraviolet–visible (UV-Vis) spectra were measured on a UV-3600 spectrometer (Shimadzu, Japan). UV-Vis spectrophotometer (Shimadzu BioSpec nano) was preferred to determine the concentration and purity of single-stranded oligonucleotide through absorbance measurement at 260 and 280 nm. Fourier transform infrared (FTIR) spectra were measured using a Perkin Elmer spectrophotometer in the range of 400 to 4,000 cm^−1^. X-ray diffractor (XRD) PANalytical X’Pert-Pro (0.02 °/s) was used to analyze the crystalline patterns of all samples. Field-emission scanning electron microscopy (FESEM) was collected using a JSM-7610FPlus microscope (JEOL) along with energy-dispersive x-ray spectroscopy (EDS) attachment to determine the morphological features along with details of elements that are present in the sample. FEI Tecnai G2 S-Twin transmission electron microscope (TEM) was utilized to obtain the high-resolution images of samples along with selected-area electron diffraction (SAED) patterns. Fluorescence spectra were obtained using solid black 96-well plates on SpectraMax M5 multi-mode reader (Molecular Devices, San Jose, USA). The performance of the proposed biosensor for the detection of the lowest concentration of Hg^2+^ is compared with ICP-MS (Agilent 7700 series).

### Synthesis of CuNC-encapsulated Zr-MOF

Synthesis details of CuNCs and Zr-MOF are provided in Sections S1.1 and S1.2, respectively. CuNCs@Zr-MOF composite was synthesized through in situ “bottle around the ship” methodology. Briefly, 1 ml of synthesized CuNCs was poured to the aqueous solution of zirconyl chloride (0.3 g) in water. Organic linkers, 1,2,4,5-benzenetetracarboxylic acid (btec) and 2,4,6-tris(4-pyridyl)-1,3,5-triazine (tpt), were dissolved in 10 ml of water and ethanol, respectively. The mixture was allowed to stir for 30 min. An equilibrated solution of zirconia and CuNCs was poured rapidly into an equilibrated solution of organic linkers. Afterward, formic acid (1 ml) was added into the above solution. The contents were kept at stirring for 2 h and later transfer into a 50-ml steel autoclave bomb at 120 °C for 72 h. To recover the resultant product, centrifugation was done. After that, the product was washed with ethanol and deionized water repeatedly. The sample was dried under vacuum at 70 °C for overnight.

### Preparation of CuNCs@Zr-MOF-tagged aptamer signal probe

To prepare the purified single-stranded EAD2 aptamer stock solution (100 μM), nuclease-free water was preferred, and the solution was further stored at −80 °C. A one-step 1-ethyl-3-(3-dimethylaminopropyl)carbodiimide (EDC)–*N*-hydroxysuccinimide (NHS) cross-linking reaction was employed for the labeling of the 5′ end of an oligonucleotide, described in literature [36]. To activate the carboxylic groups over the CuNCs@Zr-MOF’s surface, 30 mM NHS and 20 mM EDC solution were prepared in 1 ml of tris buffer (pH 7.4), and the composite was immersed in this solution for 2 h. Later, 100 μl of the aminated EAD2 aptamer (10 μM) was added to activated CuNCs@Zr-MOF solution and incubated overnight (18 h) at 4 °C. Further, Nanosep filters were used to remove the unconjugated aptamer sequence, followed by centrifugation at 10,000*g* for 5 min. For further experiments, the final CuNCs@Zr-MOF-labeled single-stranded EAD2 aptamers were stored at 4 °C. The construction of G4 PMDNAzyme sensor is provided in Section S1.3.

### Ratiometric fluorescent sensing of Hg^2+^

The designed sensing probe was utilized in suspension form to record its optical properties (in the range of 250 to 800 nm) with an excitation wavelength of 270 nm. The changes in fluorescence of the designed biosensing probe were examined in the presence of Hg^2+^ (0 to 100 μM concentration range) in phosphate-buffered saline (PBS) buffer solution (pH 7.4). The binding of Hg^2+^ on the active sites of the EAD2 aptamers induces hybridization with cDNA fragments through T–Hg–T bonding. The above reaction system causes a decrease in fluorescence of CuNCs@Zr-MOF/PMDNAzyme (λem = 463 nm) along with an increase in fluorescence of G4 PMDNAzyme–NMM (λem = 610 nm). Finally, the fluorescence spectra were examined in the range of 250 to 800 nm (with excitation at 350 nm) utilizing 200 μl of the mixture into a 96-well fluorescence microplate. The fluorescent emission of CuNCs@Zr-MOF and NMM was recorded at 463 and 610 nm, respectively. The experiment was performed trice, and the average value of these experiments was used as the final fluorescence intensity. To evaluate the selective and specific nature of the developed probe for Hg^2+^, an interference study was also performed by adding the other metal ions (such as Cr^2+^, Co^2+^, Zn^2+^, Mn^2+^, Cu^2+^, Pb^2+^, Ni^2+^, and Cd^2+^) with a fixed concentration of 100 μM for each metal ion, keeping in view their coexistence in water resources.

### Colorimetric sensing of Hg^2+^

The chemiluminescent detection of Hg^2+^ was performed using the CuNCs@Zr-MOF/PMDNAzyme probe solution. The probe solution (200 μl) was mixed with luminol (5 μl) and H_2_O_2_ (1.3 mM) solution. After, incubating this mixture for approximately 1 min at 25 °C, it was analyzed with a luminometer. Next, different concentrations of Hg^2+^ solutions were added to the active CuNCs@Zr-MOF/PMDNAzyme solution. The magnitude of absorbance (A652) was optimized to quantify the concentration of Hg^2+^.

## Results and Discussion

### Working of dual-mode biosensor

**Fig. 1.**
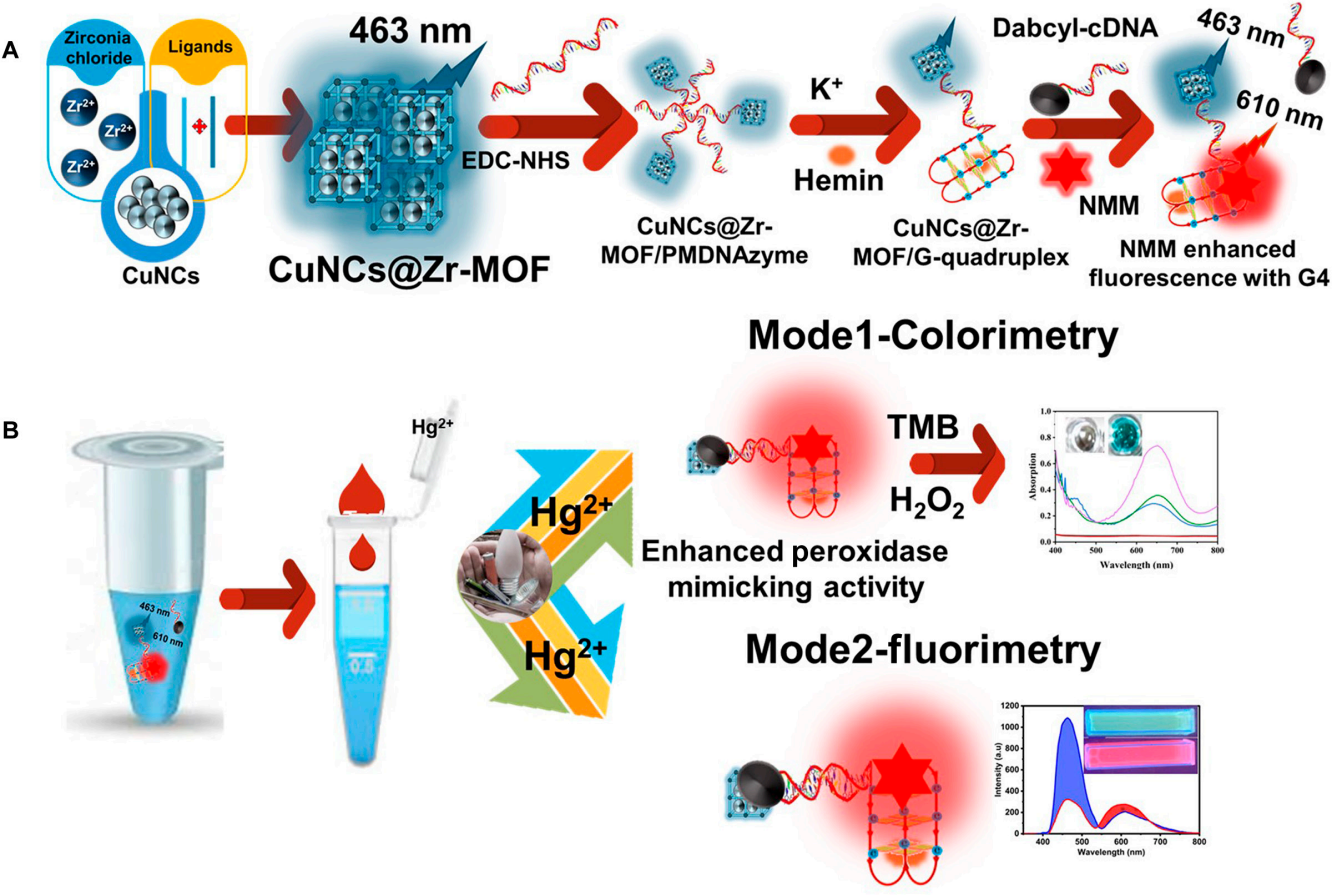
(A) Fabrication of the PMDNAzyme sensor. (B) Dual-detection mechanism in the presence of Hg^2+^ ion.

Figure 1 illustrates the principle of detection for the proposed dual G4 aptasensor. A fluorescent and colorimetric response to the presence of Hg^2+^ can be generated through PMDNAzyme anchoring on the surface of CuNCs@Zr-MOF. The aptamer strands contain a T–T mismatch vital binding site for Hg(II). T–T mismatches decrease the binding energy of DNA probes and hence are unable to form the duplex with the complementary strand. A partial hybridization with the complementary strand is possible when Hg^2+^-mediated base pairing takes place between T-residues of the G4 aptamer (T–Hg–T). Hence, it can be utilized as a recognition probe for the designing of biosensors. Hg concentration determines the amount of aptamer hybridization. To improve the detection sensitivity, ratiometric fluorescent strategy was incorporated into the aptasensor. Here, CuNCs@Zr-MOF and NMM were employed as the two fluorescent probes, while dabcyl was employed as a quencher to generate a ratiometric response signal. The significant role of the CuNCs@Zr-MOF nanoconjugate is to enhance the signaling and analytical performance of the aptasensor. However, in the presence of hemin, the aptamer forms a stable peroxidase-mimicking G4 structure. Moreover, NMM (a G4 intercalation dye) binds strongly to the G4 structure and emits the enhanced fluorescence. As a result, in the absence of target ion (Hg^2+^), dual blue/red fluorescence (corresponding to CuNCs@Zr-MOF and NMM, respectively) is obtained.

In the presence of toxic Hg^2+^, the hybridization of the CuNCs@Zr-MOF-labeled complementary strand with the dabcyl (quencher)-tagged G4 strand leads to fading of its blue fluorescence intensity because of the FRET process along with no significant effect over peak at 610 nm. Under this condition, Intensity_610_/Intensity_463_ was used as the fluorescent signal output. This variation in fluorescence intensity of the sensing probe can be optimized to quantify Hg^2+^. The complementary strands in larger amounts can be conjugated with the nanocomposite fluoroprobe to detect Hg^2+^ with excellent sensitivity. Moreover, the engineered luminescent nanomaterial strategy helps in improving the signaling of the biosensing probe. In the presence of target ions, the designed fluoroprobe induces higher changes in fluorescent signal in comparison to conventional fluorophores like organic dyes and bare QDs.

The proposed PMDNAzyme oligonucleotide probe integrates several capabilities into one oligonucleotide, including recognition, fluorescent, and colorimetric signals. The G4 aptamer binds strongly with the hemin to form the G4–hemin PMDNAzyme complex with the peroxidase-like activity. PMDNAzymes catalyzed TMB/H_2_O_2_ colorimetric reaction. The experimental results indicate that PMDNAzyme offered a higher catalytic activity in the presence of Hg^2+^ ions due to partial hybridization as compared to single-stranded PMDNAzyme, thereby greatly magnifying the colorimetric detection performance.

### Characterization of CuNC-doped Zr-MOF nanoconjugate

**Fig. 2.**
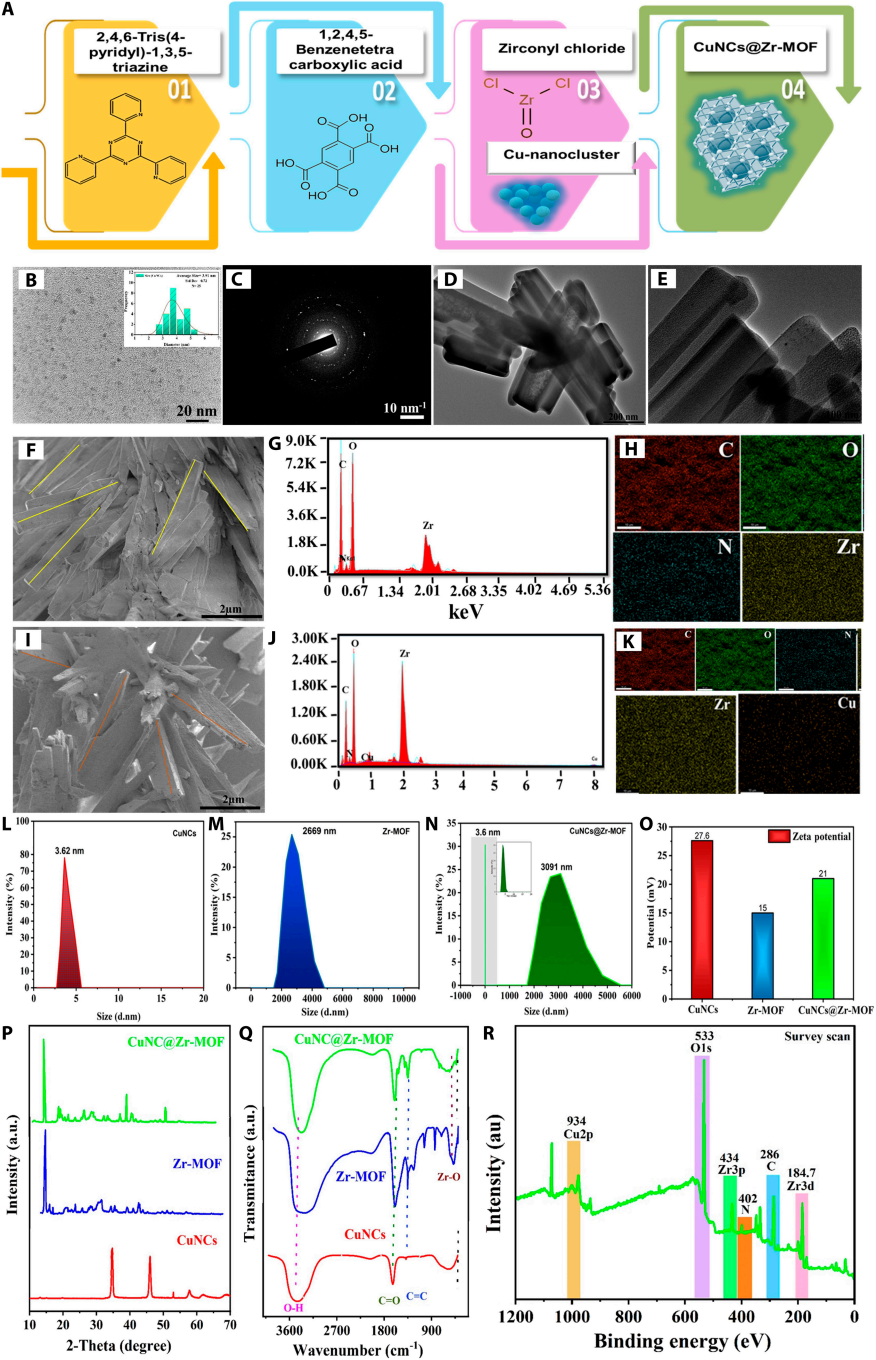
Structural and morphological characterization of CuNCs, Zr-MOF, and CuNCs@Zr-MOF nanoconjugate. (A) Schematic representation of fabrication of the highly fluorescent CuNCs@Zr-MOF nanoprobe. HRTEM images of (B) CuNCs and (C) SAED pattern of CuNCs. HRTEM images of (D) Zr-MOF and (E) CuNCs@Zr-MOF. SEM images of (F) Zr-MOF, (G) EDS line spectrum, and (H) elemental mapping of a Zr-MOF. SEM image of (I) CuNCs@Zr-MOF nanoconjugate, (J) EDS line spectrum, and (K) elemental mapping of CuNCs@Zr-MOF. DLS distribution of (L) CuNCs, (M) Zr-MOF, and (N) CuNCs@Zr-MOF. (O) Zeta potentials of all synthesized nanostructures. (P and Q) XRD and FTIR patterns, respectively, of CuNCs, Zr-MOF, and CuNCs@Zr-MOF. (R) XPS spectra of CuNCs@Zr-MOF.

Here, the high-intensity fluorescent nanoconjugate (i.e., CuNCs–Zr-MOF) was synthesized through “ship around the bottle” self-assembly methodology in order to integrate the superior fluorescence capabilities of individual entities into one nanotag for biosensing application (Fig. 2A). Carboxylated CuNCs were chosen for the high-intensity signal nanoconjugate formation due to their good chemical and optical stability. The selected 2-dimensional (2D) Zr-MOF nanostructure possessed a porous morphology with high specific surface area. Figure 2B to E shows the typical high-resolution transmission electron microscopy (HRTEM) images of CuNCs and pattern of CuNCs, Zr-MOF, and CuNCs@Zr-MOF, respectively. The HRTEM image of CuNCs displays homogeneous distribution of small spherical structures with a diameter of ~2 to 4 nm (refer to histogram, Fig. 2B, inset). The Gaussian fit for this histogram displays the maxima of diameter at 3.9 nm. The SAED pattern (Fig. 2C) confirms the crystallinity of CuNCs. The HRTEM images of Zr-MOF (Fig. 2D) show highly porous rod-like morphology, and the HRTEM image of CuNCs@Zr-MOF (Fig. 2E) clearly illustrates the density of CuNCs inside the pores and on the surface of Zr-MOF. Likewise, the FESEM micrographs (Fig. 2F and I) and EDS spectra (Fig. 2G and J) revealed that formed CuNCs@Zr-MOF maintained their monodispersity, surface morphology, and doping of CuNCs. The elemental mapping (Fig. 2H and K) revealed the doping and dense distribution of Cu (CuNCs) along with zirconium, carbon, nitrogen, and oxygen elements, indicating successful formation of the CuNCs@Zr-MOF nanocomposite. Dynamic light scattering (DLS) confirmed that the doping of CuNCs in the Zr-MOF structure slightly increases the hydrodynamic size and dispersity of the CuNCs@Zr-MOF nanoconjugate (Fig. 2l to N). Figure 2O shows the surface potential of CuNCs, Zr-MOF, and CuNCs@Zr-MOF, indicating good colloidal stability of the nanoconjugate.

Crystallographic structures of the synthesized materials were determined using x-ray diffraction (XRD), as depicted in Fig. 2P. Sharp diffraction peaks were observed at 2θ = 46.2° and 35° in CuNCs, corresponding to the planes (111) and (220) of CuO crystals (CCDC 638866) [37]. Notably, the resultant product does not possess crystallinity peaks of copper nanoparticles, which could be due to the synthesis of small nanoclusters. CuNCs diffractogram (Fig. 2P, red line) demonstrates the crystal structure with a space group of p-1. In the case of Zr-MOF (Fig. 2P, blue line), the strong diffraction peaks at 2θ = 5.4°, 8.4°, 12.8°, 16.2°, 25.2°, and 38.2° can be assigned to (002), (022), (222), (004), (224), and (006) planes, respectively (JCPDS No. 37–1492) [38]. The XRD of CuNCs@Zr-MOF (Fig. 2P, green line) exhibited identical patterns to that simulated for Zr-MOF, except the effect over the intensity of peaks due to the presence of CuNCs.

All synthesized nanostructures were further analyzed by FTIR to determine their chemical structure, as demonstrated in Fig. 2Q. Detailed information is provided in Section S2.1. Further, an x-ray photoelectron spectroscopy (XPS) analysis was performed to determine the chemical states of various elements present in the synthesized CuNCs@Zr-MOF nanocomposite (Fig. 2R) (refer to Section S2.2).

### Optical analysis of CuNCs@Zr-MOF nanoconjugate

**Fig. 3.**
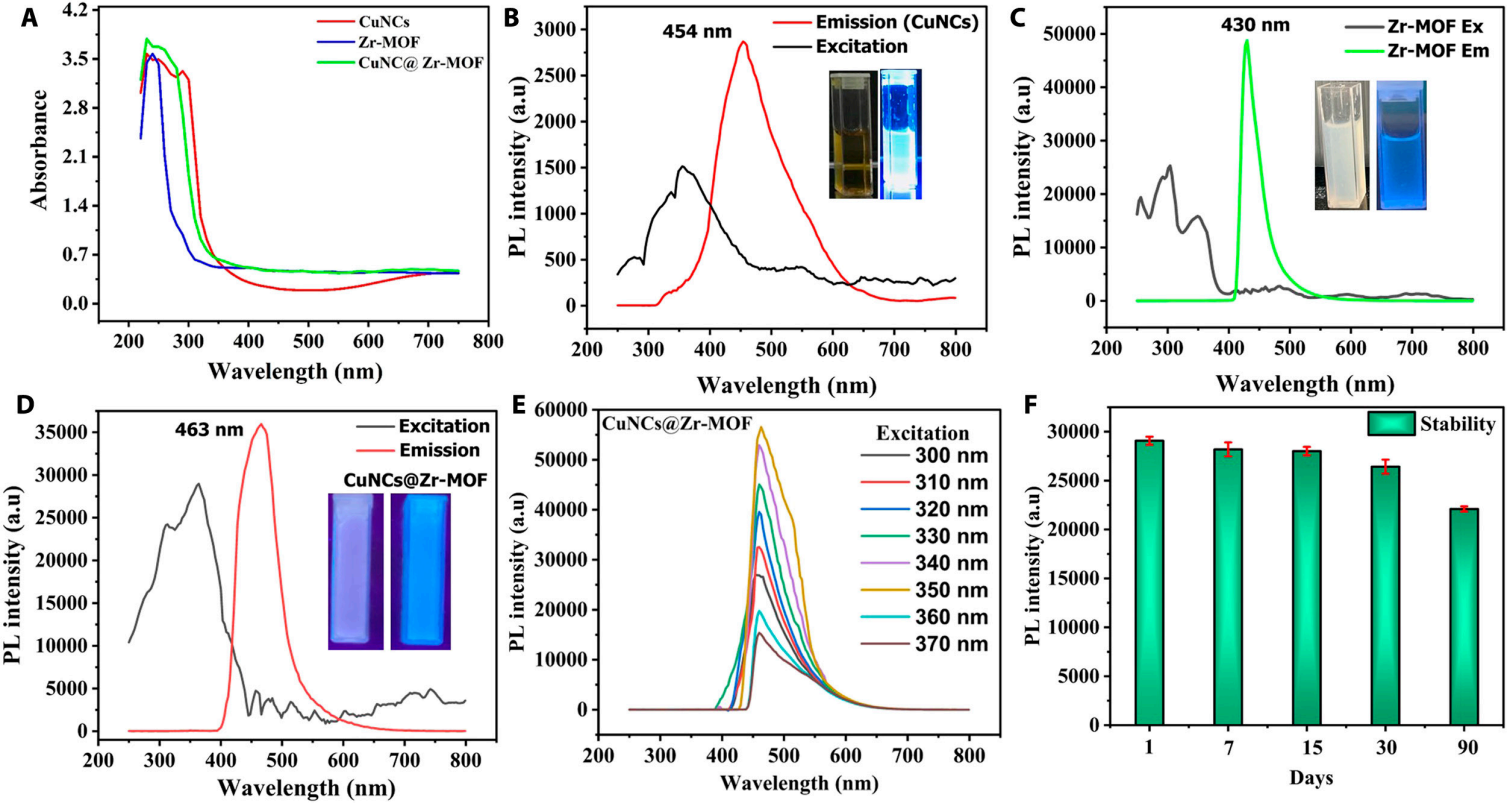
Optical characteristics. (A) UV-Vis absorption spectra. (B) CuNCs photoluminescence spectra. (C) Zr-MOF photoluminescence spectra. (D) CuNCs@Zr-MOF photoluminescence spectra. (E) Excitation-dependent emission spectra of CuNCs@Zr-MOF. (F) Photoluminescence stability of CuNCs@Zr-MOF (error bars represent 95% confidence intervals).

The absorption and fluorescence properties of CuNCs@Zr-MOF have important functional aspects in sensing applications (Fig. 3). The UV spectrum of CuNCs demonstrates three absorption peaks at 230, 250, and 290 nm, corresponding to the quantum confinement, molecular-like behavior, and excitonic features, respectively (Fig. 3A, red line) [39,40]. This is significantly different from the characteristic absorption peak (650 nm) due to surface plasmon resonance effect in Cu nanoparticles, suggesting the conversion to smaller NCs. Further, the spectrum of Zr-MOF (Fig. 3A, blue line) displayed dominant absorption peak at 240 nm [41] and weak band at 290 nm corresponding to n–π* and π–π* transitions, respectively, due to tpt and BTEC organic ligands [42,43]. Moreover, the absorption around 410 nm can be attributed to transition of e^−^ from O_2_ atoms in BTEC to Zr metal ions. The CuNCs@Zr-MOF (Fig. 3A, green line) nanocomposite had characteristic peaks of both CuNCs and Zr-MOF. The peak positions centered at 230 and 260 nm can be designated to the presence of CuNCs transitions, while a broad peak at 410 nm is similar to the parent compound (i.e., Zr-MOF). There is a peak shift from 290 to 330 nm in CuNCs@Zr-MOF because of the interaction between CuNCs and Zr-MOF.

The luminescent properties of the synthesized nanoconjugate, CuNCs@Zr-MOF, were further characterized by a fluorescence spectrophotometer, as shown in Fig. 3B. The spectrum of CuNCs exhibited fluorescent emission centered at 454 nm upon excitation at 350 nm. It is a transparent liquid under natural light and shows a bright blue color fluorescence upon excitation with UV lamp at 365 nm (Fig. 3B, inset). The intense peak at 454 nm can be ascribed to the band edge effect [44]. The aqueous dispersion of Zr-MOF (Fig. 3C, inset) also offers similar blue luminescence centered at 430 nm at excitation of 350 nm (Fig. 3C). This luminescence behavior can be accredited to the ligand–ligand π−π* transitions. Besides this, the nanoconjugate CuNCs@Zr-MOF (Fig. 3D) exhibited high-intensity blue luminescence with characteristic emission centered at 463 nm after excitation at 350 nm. A significant red shift in the fluorescent emission peak of the nanoconjugate relative to Zr-MOFs possibly reflects the encapsulation of CuNCs. In this work, we have synthesized fluorescent materials with wide range of emission (400 to 600 nm) for the first time through the guest–host doped strategy. In addition, the fluorescence intensity of CuNCs@Zr-MOF was found higher than that of its counterparts, indicating the positive effect of conjugation on their luminescent properties. Host molecules Zr-MOF exhibit synergies with guest CuNCs in excited states, which confirm the potential turn-off and turn-on fluorescence switching of CuNCs@Zr-MOF for sensing Hg^2+^. The fluorescence emission spectra of CuNCs@Zr-MOF with respect to different excitation wavelengths were also examined for studying the changes in fluorescence intensity (Fig. 3E). The fluorescence intensity of the nanoconjugate was found excellent at the 350-nm excitation wavelength. The spectra also showed the red shift in fluorescence wavelengths due to the nonhomogeneous size of CuNCs, encapsulated inside the Zr-MOF. The photo-stability test of the nanoconjugate in buffer solution over 90 days indicates the repeatable and stable fluorescence emission for CuNCs@Zr-MOF.

### Optimization and characterization of CuNCs@Zr-MOF/PMDNAzyme sensing probe

**Fig. 4.**
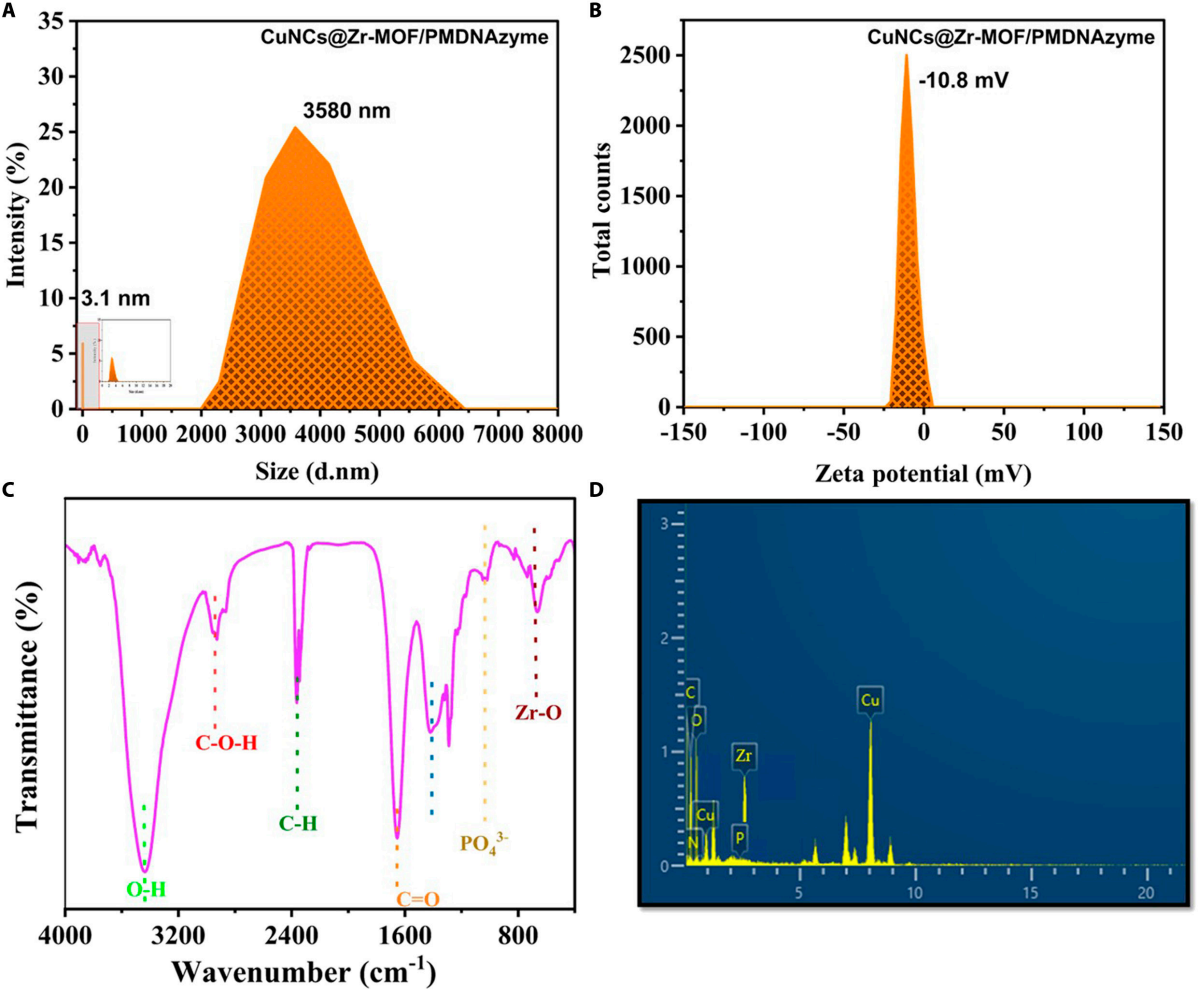
Characterization of CuNCs@Zr-MOF/PMDNAzyme sensing nanoprobe. (A) Particle size analysis. (B) Zeta potential. (C) FTIR. (D) EDS spectrum.

The carboxyl group associated with CuNCs@Zr-MOF offered the active anchoring sites for NH_3_-PMDNAzyme sequence and yielded the CuNCs@Zr-MOF/PMDNAzyme hybrid. The successful labeling of DNAzyme sequence was verified by DLS and FTIR measurements. The hydrodynamic particle size of CuNCs@Zr-MOF/PMDNAzyme was found slightly bigger (i.e., 3580 nm) than the nanoconjugate, demonstrating the successful conjugation of PMDNAzyme with CuNCs@Zr-MOF (Fig. 4A). Further, the zeta potential of CuNCs@Zr-MOF (i.e., 21 mV) becomes −10.8 mV after conjugation with negatively charged PMDNAzyme (Fig. 4B). In the FTIR spectrum (Fig. 4C), this conjugation of CuNCs@Zr-MOF with PMDNAzyme induces additional vibrational peaks at 1046 characteristic of DNA in addition to parent peaks of CuNCs@Zr-MOF [45]. These peaks can be assigned to the symmetric stretching vibrations of PO_4_^3-^ (P═O and P═O) and bases in oligos. Therefore, all these characterizations confirm the fabrication of functional fluorescent probes by labeling PMDNAzyme with CuNCs@Zr-MOF. EDS characterization confirmed the presence of N, P, and Zr elements in the CuNCs@Zr-MOF/PMDNAzyme probe (Fig. 4D). All these physical characterizations provided a clear view of the conjugation of the nanoconjugate to G4 PMDNAzyme. Further, optimization of the G4 system is provided in Section S2.3.

### Dual-mode sensing of Hg^2+^ ions with the fabricated sensor

#### Colorimetric sensing

**Fig. 5.**
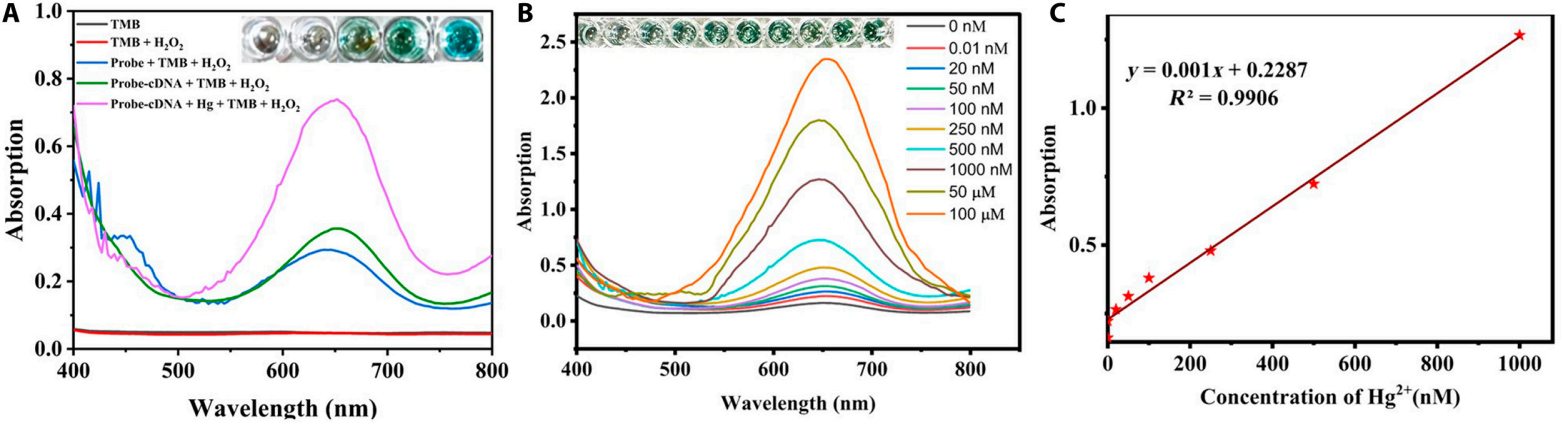
Colorimetric responses. (A) Absorption spectra of TMB, TMB/H_2_O_2_, probe/TMB/H_2_O_2_, probe-cDNA/TMB/H_2_O_2_, and probe-cDNA/Hg/TMB/ H_2_O_2_. (B) Absorption at 652 nm for the different concentrations of Hg^2+^. (C) Linear fitting curve.

On the basis of the excellent peroxidase-mimicking property of the fabricated system, a colorimetric biosensor was fabricated for the detection of Hg^2+^. Fan et al. [25] confirmed an axial high-spin ferric heme in hemin. Upon binding of G4 DNA to hemin, the peroxidase activity mainly results from guanine to heme iron coordination. In the presence of Hg^2+^, stable duplex formation can be observed due to the combination of cDNA with a part of G4 DNA. This results in some reorganizations in the 3D structure of the probe, along with enrichment of the catalytic activity of PMDNAzyme. The peroxidase activity of the synthesized probe (in the presence of Hg^2+^) was analyzed using the chromogenic TMB substrate along with H_2_O_2_. The PMDNAzyme G4 structure catalyzes the oxidation of TMB (colorless) to a bluish green solution (oxTMB), confirmed by an absorption peak at 652 nm. However, in the presence of Hg^2+^, the absorption value at 652 nm was found higher in the case of CuNCs@Zr-MOF/PMDNAzyme-Hg-cDNA (Fig. 5A). In order to demonstrate the effectiveness of CuNCs@Zr-MOF/PMDNAzyme-cDNA for the quantitative analysis of Hg^2+^, the relationship between analyte concentration and the magnitude of the UV absorbance was studied. Under optimal conditions, the concentration of Hg was found to be proportional to the intensity of blue color (Fig. 5B). Moreover, a fine linear response was observed in the 0 to 100 μM concentration range of Hg (Fig. 5C). Considering the signal to noise ratio (S/N = 3) formula, the limit of detection (LOD) was calculated as 36.3 nM/l. Therefore, this approach offered a wide linear detection range along with desirable sensitivity.

#### Fluorimetric sensing

**Fig. 6.**
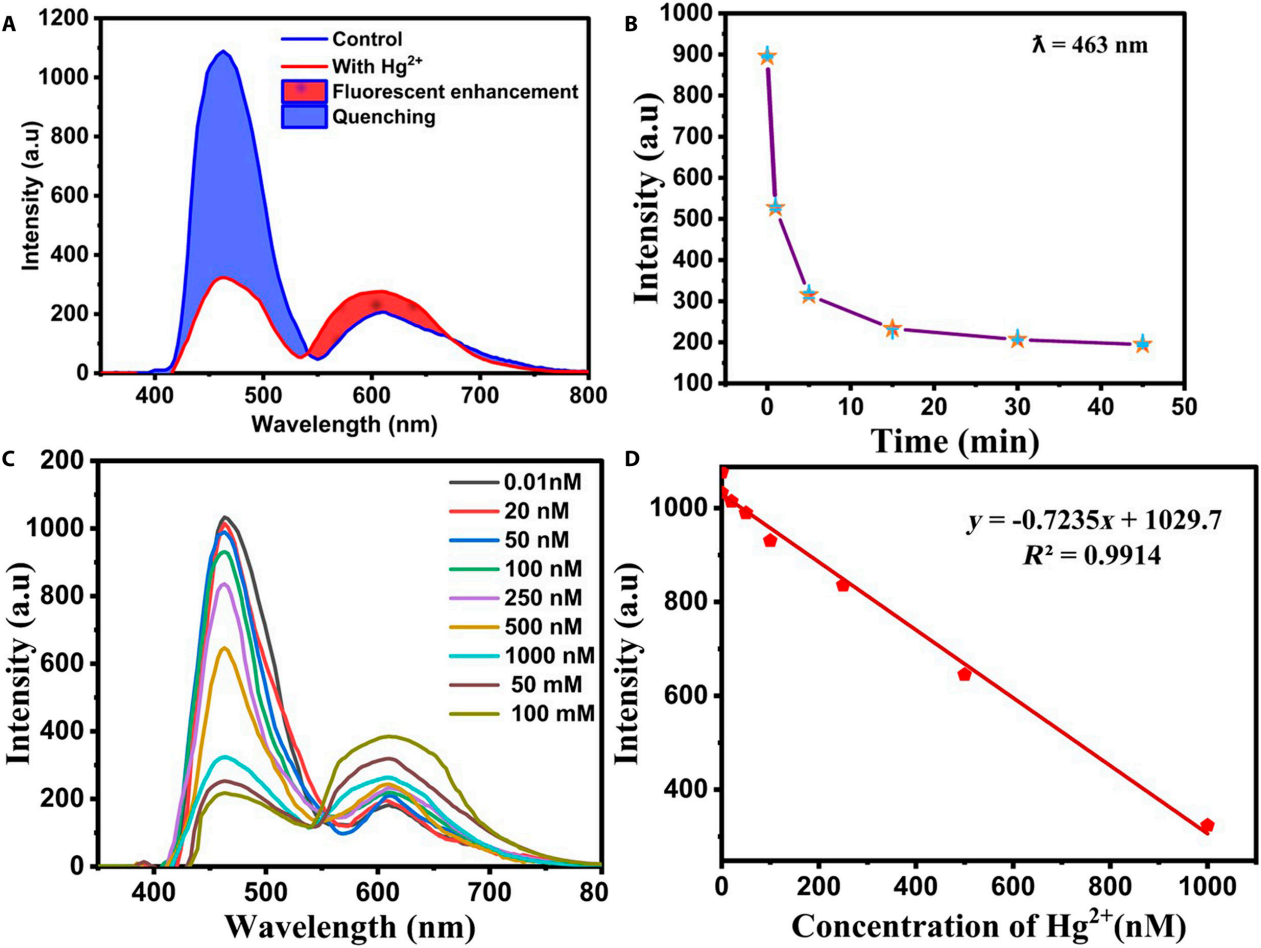
Fluorescence response of CuNCs@Zr-MOF/PMDNAzyme-cDNA sensor. (A) Illustration of the ratiometric fluorescence. (B) Effects of response time. (C) Fluorescence intensity at 463 and 610 nm for the different concentrations of Hg^2+^. (D) Linear calibration curve.

Quantitative analysis of Hg^2+^ ions in water was performed using the ratiometric system (i.e., CuNCs@Zr-MOF/PMDNAzyme–NMM–cDNA) under the conditions described above. The developed functional nanoprobe “CuNCs@Zr-MOF/PMDNAzyme” had a dual emission (blue and red) at 463 and 610 nm. The specific binding of the nanofluorophore-conjugated quadruplex probe to Hg^2+^ causes the hybridization of mismatched dabcyl-cDNA through T–Hg^2+^–T base pairing. Here, the FRET process was initiated due to the proximity of the energy donor and acceptor, causing the fluorescence quenching of CuNCs@Zr-MOF. Moreover, the interaction of Hg^2+^ with the sensing probe, governing the confirmation of G4, leads to the fluorescence enhancement due to NMM. Hence, the synthesized probe showed ratiometric fluorescence on interaction with Hg^2+^. Figure 6A confirms the decreased fluorescence intensity curve at 463 nm and enhancement at 610 nm. Therefore, the presence of Hg^2+^ causes considerable changes in the fluorescence of the biosensing probe, resulting in red fluorescence states. The FRET system (CuNCs@Zr-MOF/PMDNAzyme–NMM–cDNA) was also analyzed for incubation period upon addition of Hg. A maximum quenching of blue luminescence intensity was observed up to 5 min of Hg^2+^ incubation at room temperature (Fig. 6B). The effectiveness of the quenching strand for energy transfer in between the donor (CuNCs@Zr-MOF) and the acceptor (dabcyl) was investigated by mixing CuNCs@Zr-MOF/PMDNAzyme-cDNA with Hg^2+^ ions.

Further, the sensitivity of this biosensor was determined by examining the degree of quenching with increasing concentration of Hg^2+^ (0 to 100 μM) (Fig. 6C). Under optimal experimental conditions, the concentration of Hg was found proportional to the quenching at 463 nm. The linear relationship between the increasing Hg^2+^ concentration and the quenching was obtained for the concentration range from 1 to 1000 nM (Fig. 6D). The Stern–Volmer curve regression equation, *Y* = −0.7235*X* + 1029.7, with a reliable correlation coefficient *R*^2^ = 0.9914 confirms a good linear relationship. In particular, the Stern–Volmer constant (*K*_sv_) was estimated to be 7.2 × 10^–10^ M^–1^ for Hg^2+^ at 463 nm. The fabricated biosensing probe offered low LOD of 0.59 nM.

The comparative analysis of performance of the CuNCs@Zr-MOF/PMDNAzyme-cDNA nanosensor for Hg^2+^ detection to those reported previously is provided in Table. There are a number of limitations in these previously reported literature on Hg sensors, such as poor selectivity, poor sensitivity, or poor specificity. First, all the paper’s focuses on a single detection system using organic fluorophores or nanomaterials do not have sensitivity and photostability. As part of this study, the authors gave preference to novel research such as the synthesis of a high-intensity ratiometric fluorophore (CuNCs@Zr-MOF), focusing on improving the efficiency of fluorescence signaling. Moreover, a nanozyme or G4 has many advantages over natural enzymes, such as good biocompatibility and multifunctionality, through atomic-level materials engineering. Adding two detection signals to a single measurement system improves performance not only through reduced assumptions but also by increasing application flexibility, improving accuracy, and increasing linear range.

**Table.** Limits of detection for already reported biosensors for Hg^2+^

S.No.	Nanomaterial	Technique	Detection limit	Reference
1.	Salic_OVA-AuNCs nanoprobe	Fluorescent	0.13 nM	[32]
2.	Dual-carbon dots	Fluorescent	5.3 nM	[46]
3.	Polystyrene (PS)-AgNPs and Ps-AuNPs/aptamer	Colorimetric and electrochemical	5 and 0.5 ppm	[47]
4.	Benzothiazole-based fluorescent probe	Fluorescent	2.85 nM	[48]
5.	Coumarine monomer	Fluorescent	20 nM	[49]
6.	Carbon dot/AgNPs	Raman spectroscopy	0.01 nM	[50]
7.	Cys-AgNPs	Colorimetric	45 nM	[51]
8.	Apt-AuNCs	Fluorescent	0.016 nM	[52]
9.	Apt-GO	Fluorescent	0.0001 nM	[53]
10.	CuNCs@Zr-MOF-PMDNAzyme	Fluorescent and colorimetric	0.59 and 36.3 nM	This work

### Specificity of the fabricated sensor for cadmium

**Fig. 7.**
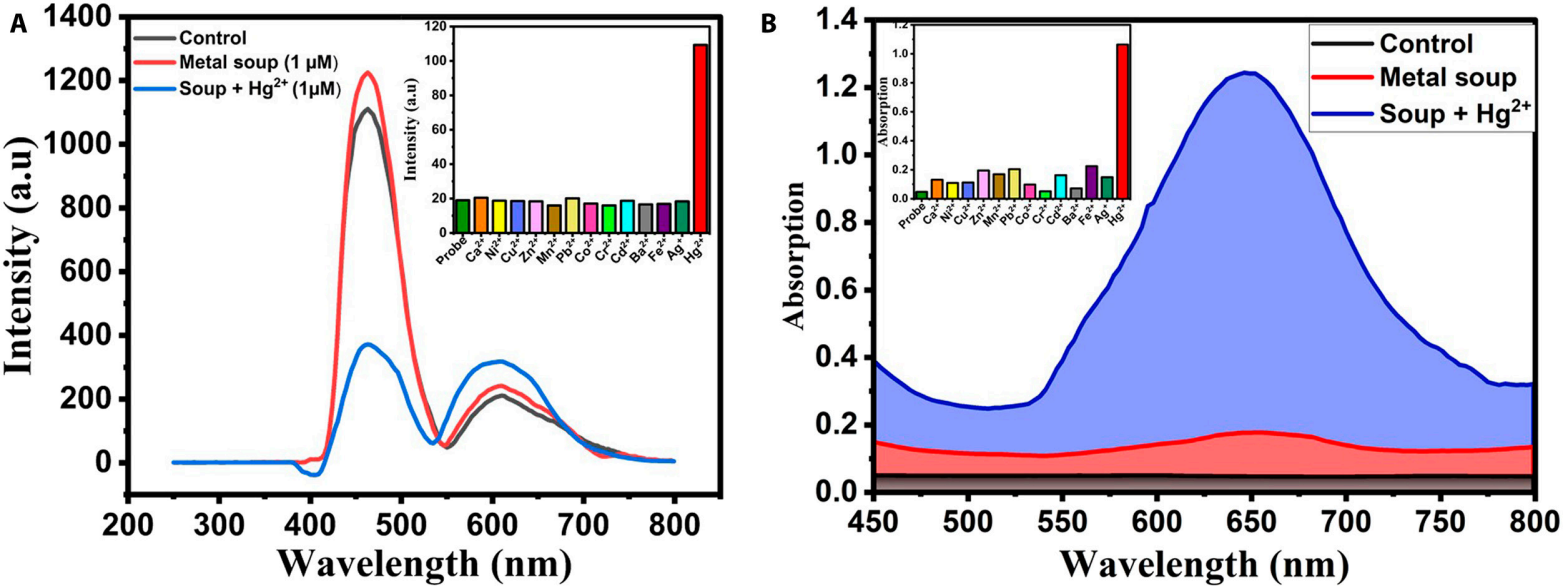
Specificity of CuNCs@Zr-MOF/PMDNAzyme-cDNA system toward Hg^2+^. (A) Fluorescent mode [inset: relative fluorescent intensity (*I*_610_/*I*_463_*100) against different metal ions]. (B) Colorimetric mode (inset: absorbance against different metal ions).

The specificity of the CuNCs@Zr-MOF/PMDNAzyme-cDNA system toward Hg^2+^ was examined in the presence of different metal ions (e.g., Cr^2+^, Co^2+^, Zn^2+^, Mn^2+^, Cu^2+^, Pb^2+^, Ni^2+^, Ca^2+^, Ba^2+^, Fe^2+^, Ag^+^, and Cd^2+^) that can potentially interfere during the detection of Hg^2+^. These metal ions were introduced as a metal soup and individually in PBS buffer. A concentration of 1000 nM of each of these metal ions was mixed with Hg (1 μM) under optimal experimental conditions. None of these metal ions caused a significant quenching of the fluorescence and enhancement of colorimetric intensity as compared to the presence of Hg^2+^ (refer to Fig. 7A and B). These results clearly highlight the high specificity of the designed dual-mode optical sensor for the detection of Hg^2+^.

Moreover, the fabricated biosensor can be restored to its original binding capacity after it has been used. This allows its use for multiple cycles of detection without any need of the PMDNAzyme probe replacement. The capture probe can be regenerated through thermal or chemical (urea) treatment for the next experiment. After the analyte is removed, the aptamer returns to its original folded state, and thus, easy regeneration of the sensor makes it more robust.

## Conclusion

Here, a dual-signal biosensor based on the colorimetric and fluorescent approach is developed for the detection of Hg^2+^ in aqueous solutions. To achieve this, a novel biosensing probe was synthesized utilizing CuNCs, Zr-MOF, PMDNAzyme, NMM, and cDNA. The CuNCs@Zr-MOF composite, synthesized via in situ “bottle around the ship” methodology, exhibited excellent fluorescent emission at 463 nm upon excitation at 350 nm. This composite in combination with the NMM fluorophore offered dual-fluorescence emission at 463 and 610 nm. The presence of Hg^2+^ causes the hybridization of PMDNAzyme and cDNA, resulting in ratiometric fluorescent changes for the detection of these metal ions. Moreover, this biosensing probe also offers colorimetric detection of Hg^2+^ due to the peroxidase-mimicking activity of G4 PMDNAzyme in the presence of hemin. This dual-signal sensor offered the rapid screening and quantitative analysis with LODs of 0.59 and 36.3 nM in the case of fluorescent and colorimetric approaches, respectively. The sensitivity of this dual-mode sensor was found higher without increasing the testing time (i.e., 5 min) in comparison to already reported biosensors. This dual-mode platform can serve as a user-friendly and affordable tool for rapid sensing of Hg^2+^. In addition to providing a reliable method for Hg detection, the developed strategy opens up new research avenue for the development of dual-signal sensing systems with high accuracy and reliability.
